# Envisioning gamification in anesthesia, pain management, and critical care: basic principles, integration of artificial intelligence, and simulation strategies

**DOI:** 10.1186/s44158-023-00118-2

**Published:** 2023-09-11

**Authors:** Marco Cascella, Andrea Cascella, Federica Monaco, Mohammed Naveed Shariff

**Affiliations:** 1 Department of Anesthesia and Critical Care, Istituto Nazionale Tumori-IRCCS, Fondazione Pascale, Via Mariano Semmola, 53, 80131 Naples, Italy; 2 Freelance IT Developer, Stockholm, Sweden; 3 Department of Anesthesia, ASL Napoli 1, Naples, Italy; 4 Department of Artificial Intelligence and Data Science, Rajalakshmi Institute of Technology, Chennai, India

**Keywords:** Gamification, Serious games, Education, Anesthesia, Pain medicine, Simulation, Artificial intelligence, Virtual reality, Training

## Abstract

Unlike traditional video games developed solely for entertainment purposes, game-based learning employs intentionally crafted approaches that seamlessly merge entertainment and educational content, resulting in captivating and effective learning encounters. These pedagogical methods include *serious video games* and *gamification*. Serious games are video games utilized as tools for acquiring crucial (serious) knowledge and skills. On the other hand, gamification requires integrating gaming elements (game mechanics) such as points, leaderboards, missions, levels, rewards, and more, into a context that may not be associated with video gaming activities. They can be dynamically (game dynamics) combined developing various strategic approaches. Operatively, gamification adopts simulation elements and leverages the interactive nature of gaming to teach players specific skills, convey knowledge, or address real-world issues. External incentives stimulate internal motivation. Therefore, these techniques place the learners in the central role, allowing them to actively construct knowledge through firsthand experiences.

Anesthesia, pain medicine, and critical care demand a delicate interplay of technical competence and non-technical proficiencies. Gamification techniques can offer advantages to both domains. Game-based modalities provide a dynamic, interactive, and highly effective opportunity to learn, practice, and improve both technical and non-technical skills, enriching the overall proficiency of anesthesia professionals. These properties are crucial in a discipline where personal skills, human factors, and the influence of stressors significantly impact daily work activities. Furthermore, gamification can also be embraced for patient education to enhance comfort and compliance, particularly within pediatric settings (game-based distraction), and in pain medicine through stress management techniques. On these bases, the creation of effective gamification tools for anesthesiologists can present a formidable opportunity for users and developers.

This narrative review comprehensively examines the intricate aspects of gamification and its potentially transformative influence on the fields of anesthesiology. It delves into theoretical frameworks, potential advantages in education and training, integration with artificial intelligence systems and immersive techniques, and also addresses the challenges that could arise within these contexts.

## Introduction

Drawing from the perspectives of the game designer Jane McGonigal, video games have the potential to highlight individual strengths and can assist users in achieving their objectives, boosting motivation, and fostering creativity [1]. While typical or traditional video games are designed solely for entertainment purposes, when video games are employed as instruments for learning crucial (serious) knowledge and skills, they are recognized as serious games. Therefore, game-based learning involves the utilization of intentionally designed game-based strategies, primarily focused on providing impactful educational encounters. These pedagogical techniques encompass *serious video games* and *gamification.* The core objective is to promote instructive goals, accomplished through the creation of captivating and interactive learning experiences that inspire students to successfully accomplish assignments and projects. Therefore, these techniques capitalize on human psychology’s affinity for achievement, competition, and intrinsic motivation, employing the principles of games to encourage participation and enhance user experiences [2, 3].

Despite the different definitions proposed, according to Deterding et al. [4] the term gamification more properly refers to a dynamic approach that involves the seamless integration of various gaming elements into a context that, under ordinary circumstances, might not be associated with traditional gaming activities. Although a common point of convergence is the presence of simulation elements, unlike games, gamification approaches should not implicate the intention of creating a game. In other words, gamification does not necessarily rely on video games. Activities can be “gamified” by simulating game design mechanics and narratives in an analogous manner [5, 6].

A key element in the advancement of game-based learning lies in the application of diverse artificial intelligence (AI) methodologies. The convergence of these two transformative technologies constitutes a potent synergy. Nevertheless, the spread and evolution of serious video games and gamification take divergent routes. In healthcare, the widespread acceptance of serious games has been hindered by different obstacles. The significant production costs and intricate design complexities of health games, comparable to professional entertainment games, limit their market growth. Moreover, the reliance on dedicated devices and specific time commitments for gameplay clashes with users’ varying access to technology, daily routines, and busy schedules, posing challenges to seamless integration into their lives. On the contrary, by infusing elements such as competition, rewards, challenges, and interactive experiences into domains outside of the gaming realm, gamification can effectively transform routine tasks into engaging and captivating experiences, without a significant commitment of resources [7]. Therefore, over the last decade, the medical field has seamlessly integrated gamification elements into its educational platforms [8–11].

The assimilation of gamification in the fields of anesthesia, pain medicine, and critical care offers a noteworthy pathway. Its interactive, dynamic, and notably effective potential holds the capacity to enhance learning, refine both technical and non-technical skills, and contribute to overall progress. For training purposes aimed at acquiring specific competencies, various approaches have been employed. For example, researchers developed a 3D prototype of a virtual simulation environment for neuraxial anesthesia training, and integrated gamification elements to enhance motivation and enrich the learning experience [12]. An Italian research team has implemented the FantaTraining® app (developed by Brain Refresher Lab), which emulates a football league to enhance the educational experience of anesthesia trainees enrolled in an online obstetric anesthesia course [13]. Beyond its primary role in skill and task enhancement, the synthesis of gamification with a gratifying and immersive encounter holds the promise of harnessing fundamental motivational factors, promoting deep engagement, and furnishing real-time feedback mechanisms. These features are of paramount importance in a discipline where personal skills, human factors, and the influence of stressors play a significant role in daily work activities. Gamification can be also useful for patient education, where its innovative potential can be employed to augment comfort, compliance, and understanding. For instance, available evidence indicates that engaging in an immersive experience before surgery might effectively decrease preoperative anxiety and improve perioperative compliance in children [14].

This narrative review explores the multifaceted dimensions of gamification and its potentially transformative impact on the specialty of anesthesiology. It delves into theoretical constructs, potential educational and training benefits, integration with AI systems and immersive modalities, and addresses the challenges that may emerge within these processes.

## Basic principles of gamification

The purpose of gamification is to involve individuals who may initially lack the drive (internal motivation) for change by presenting external rewards (external motivation), thus boosting intrinsic motivation. These strategies encompass establishing objectives, delivering feedback, reinforcement, and facilitating social interactions. Hence, the fundamental factors contributing to the success of gamified systems revolve around the three F’s: Fun, Friends, and Feedback.

**Fig. 1:**
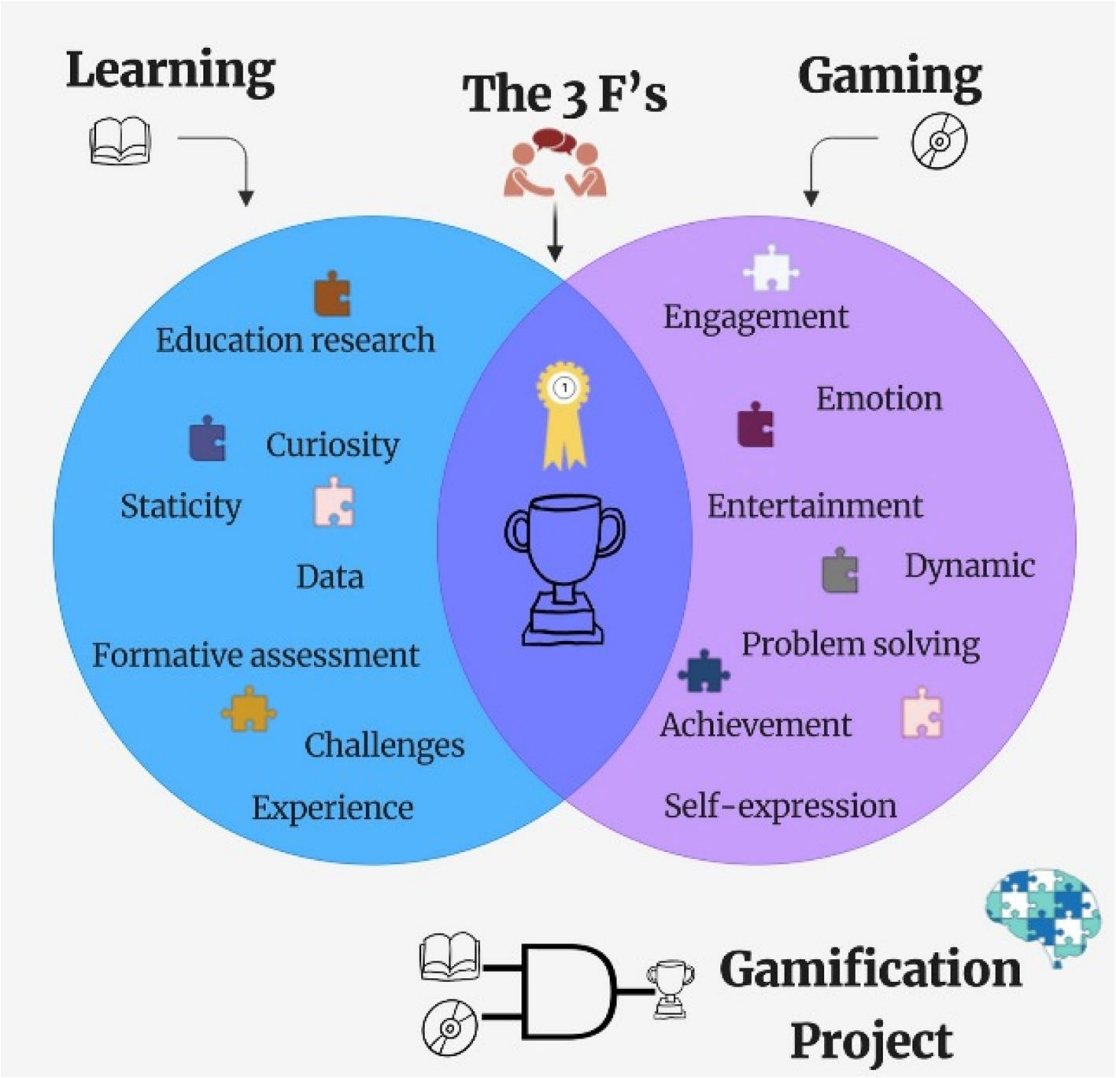
Boolean cognitive gamification. Gamification techniques prioritize user engagement within the learning process. The relationship between learning and gaming is inherently symbiotic, giving rise to a pragmatic partnership that surpasses their apparent differences. These two spheres share a foundational base built on common elements (puzzle pieces). The intersection is epitomized by the three F's: Fun, Friends, and Feedback, effectively bridging the gap between structured educational pursuits and the lively realm of dynamic recreational experiences. The output is the gamification process (see the Boolean AND symbol and the puzzle brain). In the context of gamification, a Boolean perspective could involve simplifying elements within a gamified system into binary states that, for instance, can be easily categorized as either “completed” or “not completed,” “achieved” or “not achieved,” “engaged”, or “not engaged”

From a Boolean logic [15], useful for analyzing complex situations by breaking them down into simpler components and evaluating them based on their fundamental properties, learning and gaming are inherently symbiotic, forming a pragmatic partnership that transcends their apparent dichotomy. These two domains share a foundational bedrock characterized by common elements such as challenges, achievements, and engagement. The intersection bridges the realm of structured educational endeavors with the vibrant world of dynamic recreational pursuits. In other words, this convergence accentuates the potential synergies that exist between the acquisition of knowledge and enjoyment (Fig. 1).

Notably, gamification techniques place the user at the center of the learning process [16]. Participants become the architects of their own learning, acquiring knowledge through firsthand experiences. This approach enhances their motivation, enabling them to retain information in a meaningful and enduring manner. Since the learning process is typically intertwined with an enjoyable and rewarding experience, the integration of knowledge acquisition and participation in a positive and pleasurable interaction not only enhances understanding but also fosters a sense of satisfaction and enthusiasm for further experience [17].

The underlying philosophy and functioning are very interesting. Gamification harmoniously merges principles from psychology and technology, utilizing game mechanics and dynamics as strategic elements that prompt specific behaviors. A rich array of diverse game mechanics can be implemented, each founded on distinct elements. This gamification toolkit includes points and badges, leaderboards, challenges and quests, narrative and storytelling modes, progression and levels, levels, mission, with or without collaboration and social interaction, avatars (patients or team members) and customization, rewards and incentives, feedback and progress tracking, as well as simulation and real-world applications. On the other hand, game dynamics encompass the emotions, actions, and aspirations embedded within game mechanics. Dynamics include features such as competitive leaderboards, collaborative team missions, community interaction, sets of badges, and unexpected rewards upon unlocking new challenges. They create an environment that captivates, motivates, and empowers users to actively participate and achieve desired outcomes. Concerning dynamics rationale, these processes leverage intrinsic human desires, such as achievement, competition, mastery, and social interaction, to promote engagement and participation. The memorable and immersive experiences created by game dynamics boost information retention and recall, making the learning or engagement process more effective. Ultimately, the synergy between game dynamics and mechanics nurtures commitment, propelling users toward the establishment of habitual interactions [18].

Different gamification strategies can be customized and combined to suit specific learning objectives, target audiences, and desired outcomes, making the learning process more engaging, enjoyable, and effective (Table 1). For example, the challenge-based strategy sets forth tasks and puzzles that stimulate critical thinking and problem-solving, enhancing participants’ cognitive engagement and skill development [19]. This method can be combined with the immersive strategy, creating an engrossing narrative context that immerses users within the gamified learning experience [20]. By weaving storytelling elements into the educational process, learners become active participants in a dynamic and captivating journey, fostering deeper comprehension and retention of knowledge.

**Table 1:** Gamification mechanics, dynamics, and strategies

	**Functioning**	**Rationale**
**Game mechanics**		
Points and badges	Straightforward gamification methods where participants earn points or badges for completing tasks or achieving milestones.	Capitalizes on the sense of achievement and progression, encouraging users to continue their engagement.
Leaderboards	They rank participants based on their performance, creating a competitive environment that spurs users to strive for the top position.	Taps into the natural human desire for recognition and status.
Challenges and quests	Incorporating challenges, missions, or quests adds an element of adventure and accomplishment to the learning process.	Participants undertake specific tasks or objectives, receiving rewards upon completion.
Progress bars and levels	Similar to video games, participants progress through different levels as they achieve specific goals or demonstrate competence.	Advancing to higher levels can provide a sense of accomplishment and drive continued engagement.
Rewards and Incentives	It offers tangible rewards, such as discounts, certificates, or tangible items.	Motivates participants to actively participate and excel in the learning experience.
Feedback and Progress Tracking	Regular feedback on participants' progress, strengths, and areas for improvement.	They keep participants informed and motivated to continuously enhance their performance.
**Game dynamics**		
Competitive leaderboards, collaborative team missions, and community interaction, sets of badges, and unexpected rewards upon unlocking new challenges	Dynamics create an environment that captivates, motivates, and empowers users to actively participate and achieve desired outcomes.	Leverage intrinsic human desires, such as achievement, competition, mastery, and social interaction, to enhance engagement and participation.
**Strategies**		
Quizzes	Can be used for assessing learning skills as well as the efficacy of other gamification tools.	Facilitate interactive assessments and provide immediate rewards.
Narrative and storytelling	Immersing participants in a compelling narrative can make the learning experience more engaging and memorable.	Users become active participants in a storyline, interacting with characters and making choices that impact the outcome.
Avatars and customization^a^	Allowing users to create and customize their avatars or digital personas adds a personal touch to the learning process.	Fosters a sense of ownership and connection with the content.
Collaboration and social interaction	Incorporates social elements, such as team-based challenges, collaborative tasks, or peer-to-peer interactions.	Enhances engagement through social dynamics and a sense of community.
Simulation and real-world application	Real-world scenarios or simulations that allow participants to apply their knowledge and skills in practical contexts.	Enhancement of the relevance and applicability of the learning experience.

*Legend*: ^a^Patients or team members

The social-based gamification strategy revolves around the principles of competition and collaboration. It taps into the inherently social nature of humans, leveraging elements like leaderboards, achievements, and collaborative challenges to drive engagement and motivate learners to excel. This approach not only encourages healthy competition but also encourages teamwork skills and cooperation among participants, fostering a sense of community and shared accomplishment [18].

Quizzes are a cornerstone element of gamification as the quick reward mechanism contributes to a positive learning experience. Due to their accessible and versatile nature, they represent an extensively adopted modality. In gamified settings, quizzes serve as interactive assessments that engage participants while simultaneously reinforcing their understanding of specific topics or concepts. For example, within the context of obstetric anesthesia training, the FantaTraining ® app compelled participants to respond to quizzes covering lesson topics in a competitive format against other trainees [13].

Since quizzes are just one side of this multifaceted landscape, they can be combined with other options, offering participants the invaluable opportunity to assess and validate their understanding across various platforms, ranging from user-friendly web interfaces to engaging mobile applications.

### Education and training purposes, and limitations

A fundamental aim of health professional education is to develop self-reliant advanced practitioners who possess the capacity to make independent decisions within their specialized practice area, such as emergency care [21]. Thus, the field of education has undergone a profound and captivating transformation with the infusion of gamification. No longer confined to the traditional constraints of textbooks and lectures, the process of learning has blossomed into a dynamic, interactive, and immersive experience. Leveraging the magnetic appeal of game elements, digital platforms have succeeded in kindling a heightened sense of student engagement. Moreover, through the implementation of gamified assessments, assignments, and quizzes, users are seamlessly drawn into a vibrant realm of knowledge acquisition [22]. Language learning is a typical example of a gamification application [23].

The impact of this revolution has extended to the medical field, where diverse game-based learning strategies for medical education have been proposed [24]. Gamification approaches can be implemented, for example, for e-learning aims, especially when these are conducted asynchronously where the lack of motivation for learning is considered a serious issue [25]. Thus, gamification can be applied to various types of asynchronous e-learning models to overcome this challenge.

On these bases, the applications, and prospects, for both users and developers, are practically limitless [26]. This piece of evidence becomes especially significant when considering the potential for implementing innovative and high-impact approaches, such as immersive technologies. There is a wide array of examples available. Created for Stanford’s Department of Emergency Medicine, SonoGames is an annual, four-hour interactive competition designed to evaluate and enhance the knowledge of senior emergency medicine residents in point-of-care ultrasound (POCUS), imaging proficiency, and clinical decision-making. As highlighted by Lobo et al. [27], the participation of residents in SonoGames has more than doubled since its inception. Another interesting experience is the “Top Gun" competition for cardiothoracic surgery residents. It was demonstrated that the initial variation in skill levels among residents disappeared following six weeks of training and gamification. This suggests that residents who initially lagged behind in skill were able to narrow the gap by actively participating in self-directed training [28].

However, many aspects of gamification must be effectively addressed [5, 17, 22]. An overemphasis on competition could potentially generate stress and hinder collaboration among students and trainees, thus undermining a conducive learning environment. Moreover, while gamification may initially enhance motivation, this impact could diminish as users grow accustomed to the mechanics of the game. Over-reliance on extrinsic rewards is a significant concern. While rewards and incentives can initially boost engagement, there is a risk that participants may focus solely on earning rewards rather than the learning itself. This can lead to superficial engagement and reduced long-term motivation if the rewards are removed. Furthermore, the effectiveness of gamified systems can be influenced by cultural differences. Elements that prove effective in one cultural context might not resonate with participants from another culture, leading to reduced engagement [29]. Additionally, the various forms of gamification are a subject of ongoing debate. In this context, the types of interventions vary significantly, along with the timing of their implementation. A recent meta-analysis, for instance, revealed that educational interventions among students are particularly effective when they are of brief duration, lasting less than 1 week [30].

### Gamification and artificial intelligence

Gamification and AI are two transformative technologies that, when merged, offer a powerful synergy to enhance various aspects of learning, engagement, and problem-solving. Gamification leverages game design principles to make non-game contexts more engaging and interactive, while AI involves the development of systems that can simulate human intelligence and decision-making processes. Undoubtedly, when gamification and AI converge, several exciting possibilities emerge (Table 2).

**Table 2:** Artificial Intelligence possibilities for gamification

**Purpose**	**Process**	**Effect**
Personalized learning paths	Analyzing individual learners' strengths, weaknesses, and preferences enables the creation of customized gamified learning paths.	This tailored approach enhances engagement and maximizes learning outcomes.
Dynamic challenges	AI-powered algorithms can generate adaptive challenges based on the user's performance and progress.	This ensures that the difficulty level remains optimal, fostering a continuous state of engagement and improvement.
Real-time feedback	AI can provide instant feedback based on learners’ actions, helping them understand their mistakes and guiding them toward better solutions.	The immediate feedback loop enhances the learning process and promotes quicker skill development.
Content recommendation	Interactions and preferences within gamified experiences can be assessed to suggest relevant content, activities, or challenges.	This enhances user engagement and knowledge acquisition.
Natural language processing	Capabilities can be integrated into gamified interfaces to facilitate more conversational and intuitive interactions.	Makes learning and engagement feel more seamless.
Data-driven insights	AI can analyze vast amounts of data generated by gamified activities, providing educators and developers with valuable insights into learner behavior, preferences, and performance.	The data-driven approach enables continuous improvement.
Adaptive gameplay	AI can adjust the gameplay experience based on real-time user interactions and learning progress.	Users remain engaged and challenged at the appropriate level.
Emotion recognition	Analyzing users’ facial expressions and emotions during gamified activities can provide insights into emotional responses to different challenges or content.	Findings can be used to tailor experiences for emotional engagement.
Virtual coaches andassistants	AI-powered virtual characters or assistants can guide learners through gamified activities.	Availability of explanations, hints, or additional resources as needed.
Predictive analytics	AI can predict learners’ future actions or learning trajectories based on their historical data.	Helps developers create more effective gamification strategies.

Incorporating AI into gamification opens up a world of opportunities to create more personalized, adaptive, and engaging learning experiences. By harnessing the strengths of both technologies, educators, developers, and learners can unlock new levels of effectiveness and innovation in various educational and training contexts. In particular, AI has the potential to elevate personalized education, enrich learning content, streamline task automation within courses, support tutoring efforts, cultivate learner engagement, and assess training outcomes [6, 7, 20].

The convergence of gamification and AI represents a compelling synergy that has the potential to reshape various aspects of research and healthcare delivery. For example, using a creative analogy, Bignami et al. [31] drew a comparison between the role of a clinical researcher and the iconic PAC-MAN video game character. Just as PAC-MAN tirelessly devours dots and energy pills, the clinical researcher relentlessly seeks knowledge and pursues significant scientific goals. Inspired by this analogy, the authors have formulated a straightforward handbook to assist researchers engaged with AI technologies within the anesthesiology domain. Remarkably, their guide could be helpful in enhancing the efficiency of conceiving, crafting, and assessing research initiatives.

The integration and development of AI-based gamification systems can also overcome certain limitations of gamification [5]. A prominent challenge encountered with gamification pertains to its lack of sustained alignment with users’ ultimate objectives. This is precisely where the integration of AI, particularly machine learning, becomes significant, enabling the tailoring of gamification tactics to uphold heightened levels of engagement over extended periods. Furthermore, a promising opportunity arises to employ AI in forecasting future user behaviors within the gamification framework. Armed with these predictive insights, the adjustment of gamification strategies becomes viable, thereby magnifying levels of motivation.

### Data security and patient privacy

Medical data, due to its sensitivity, requires protection from unauthorized access (privacy), accuracy (safety), and constant accessibility (availability). As a consequence, balancing the engaging potential of gamification with the essential requirements of data security and patient privacy is a critical endeavor that demands careful consideration and meticulous implementation. For instance, healthcare providers interested in offering gamified applications face the complex task of maintaining patient privacy while adhering to regulations. Across the globe, different countries have established their own rules for securing medical data and safeguarding patient privacy, such as the Artificial Intelligence Act, recently issued by the European Union [32]. These strict regulations often conflict with the seamless use of gamified technologies, which rely on unfettered access to healthcare information. In addressing this challenge, data protection regulations should provide a comprehensive framework to ensure the confidentiality, integrity, and accessibility of data, especially in contexts involving personal and sensitive domains such as healthcare. Simultaneously, it is essential to develop resilient medical information technology (IT) systems capable of securely collecting and managing medical data.

## Gamification in anesthesia, pain medicine, and critical care

The potential applications of gamification within the fields of anesthesia, pain medicine, and critical care are extensive and versatile, encompassing benefits for both individual practitioners and collaborative teams. While simulation primarily targets education and training processes, advancements are dedicated to optimizing patient comfort and compliance, as well as to research aims. Moreover, a wide range of technologies have been implemented, varying in complexity. Therefore, a systematic approach to the matter is intricate, and it appears more suitable to distinctively delineate the rationale from the operational methods.

### Gamification for technical and non-technical skills: beyond task-based strategies

Usually, technical and non-technical skills exhibit a symbiotic relationship. In the specialized field of anesthesia, this synergistic link is heightened, where the proficiency of each competence complements and reinforces the other [33]. The correct execution of tasks relies on the seamless amalgamation of both groups of capabilities, tailored to the specific context. Thus, in this context, gamification offers a fascinating approach to refining technical proficiencies but also addresses non-technical aspects like communication, and collaboration. In other words, these strategies have the capacity to target and address the distinct challenges posed by the profession. The ultimate goal is to elevate the competency of professionals, thereby enhancing patient care and safety in different clinical settings.

The role of personal factors and skills in relation to the distinctive aspects of the discipline holds significant importance. Consequently, the paramount role of personal skills, human factors, and stressors impact, transcending mere task-based activities, carries profound implications for the meaningful integration of gamification. Additionally, the category of anesthesiologists is known to face a notably elevated risk of experiencing burnout [34]. This demanding medical specialty, characterized by its intricate procedures, high-stakes decision-making, and the constant need to maintain focus and precision, places significant mental and emotional strain on practitioners. Gamification’s potential to foster engagement, motivation, and skill development aligns remarkably well with these requirements. By infusing game-like elements into the learning and practice of anesthesia, pain medicine, and critical care, professionals can enhance their technical proficiencies while also refining their soft skills. Moreover, the collaborative nature of these medical disciplines resonates with the teamwork-enhancing aspects of gamification, promising to amplify group dynamics and collective effectiveness.

### Applications in anesthesia, pain medicine, and critical care

Gamification in anesthesia fields mostly involves its use within simulation scenarios. Nevertheless, while simulations provide a highly effective means of enhancing practical skills and clinical judgment, gamification can extend beyond simulations to encompass a broader range of learning methods. For instance, gamification can be integrated into didactic teaching methods, such as interactive quizzes, by presenting complex patient scenarios in a game-like format case-based scenarios, and even incorporating game-like elements into traditional lecture formats. Principal applications of gamification in anesthesia, pain medicine, and critical care are summarized in Table 3.

**Table 3:** Principal applications of gamification in anesthesia, pain medicine, and critical care

**Application**	**Uses**	**Notes**
Simulation^a^	Scenarios include airway management (e.g., intubation, difficult airway situations, and emergency airway maneuvers), regional anesthesia, CRP, trauma, intraoperative crisis management (e.g., massive bleeding, equipment failure, or allergic reactions), patient safety (e.g., identifying and managing potential safety hazards, medication errors, and adverse events), particular contexts (e.g., obstetric anesthesia), CRM principles, POCUS.	Could be integrated into immersive technologies such as VR and AR. Simulations can be used to develop and study various scenarios, drug interactions, and patient responses in controlled environments.
Obstetric anesthesia	It can be used to simulate realistic labor and delivery scenarios, allowing trainees to practice decision-making under pressure.	Dedicated apps offer powerful collaborative learning environments.
Ultrasound-guided regional anesthesia	It can be beneficial in acquiring sonoanatomy images, interpreting anatomical structures, and refining precise hand–eye coordination.	The inclusion of game-based education and learning processes in UGRA curricula is a conceivable possibility.
Game‐based distraction	Interventions based on gaming (through gamification or AR/VR) can reduce preoperative anxiety.	Strategies such as digital games, interactive and VR distractions (e.g., guided imagery and storytelling), are designed to capture the child's focus.
Pain management	For pain-related education, self-monitoring, goal setting, social support, and the instruction of self-care strategies, including stress management.	Mostly centered around app-based solutions.
Other applications:		
• Patient compliance	Gamification can be used for pre-operative training (e.g., avatar coaches) and to encourage patients to follow post-operative care instructions, including medication schedules, exercises, and follow-up appointments.	It can nurture a sense of responsibility and accountability within patients who are more likely to actively participate in their recovery process.
• Patient monitoring	Applications could be designed to monitor postoperative health status and track improvements or deteriorations in their lifestyles. It can be applied in various settings.	Games can include features such as key performance indicators and sharing achievements on social media platforms.
• Research aims	Simulation-based and gamification can be combined to study the effects of training and skill assessment, patient safety and quality improvement, and patient experience and education.	Anesthesia researchers can explore complex scenarios, gather valuable data, and develop evidence-based insights.

*Abbreviations*: *VR* virtual reality, *AR* augmented reality, *CPR* cardiopulmonary resuscitation, *CMR* crisis resource management (effective communication, leadership, situational awareness, decision-making, and teamwork), *POCUS* point-of-care ultrasound, *UGRA* ultrasound-guided regional anesthesia

*Legend*: ^a^Simulation finds applicability in all gamification processes

#### Simulation and immersive technologies

Although simulation involving standardized patients and early full-body mannequin simulators has been documented in healthcare literature since the late 1960s [35], the integration of gamification methodologies and the implementation of AI strategies have notably accelerated the progress of these learning and training approaches [36].

Simulation-based training offers several advantages in anesthesia education. Realistic scenarios often utilize high-fidelity mannequins or computer-based models to replicate various clinical situations and challenges that anesthesiologists may encounter. In these learning contexts, participants can practice critical skills and decision-making without putting patients at risk. Therefore, practitioners can refine technical skills and build confidence in handling challenging situations. Non-technical skills are also addressed through teamwork improvement, error analysis, and constructive feedback from debriefing sessions.

Simulation exercises can range from basic technical skills practice to complex multidisciplinary simulations involving anesthesia providers, surgeons, nurses, and other healthcare professionals. These simulation scenarios include airway management such as intubation, difficult airway situations, emergency airway maneuvers, cardiac arrest and resuscitation, and intraoperative crisis management such as massive bleeding, equipment failure, or allergic reactions as well as for patient safety for identifying and managing potential safety hazards, medication errors, and adverse events. Programs of simulation are employed for training in particular practice contexts such as obstetric anesthesia [37]. Moreover, simulation is also adopted for enhancing effective communication and coordination among team members and other healthcare professionals within the crisis resource management (CRM) principles (effective communication, leadership, situational awareness, decision-making, and teamwork) [38]. A special chapter on simulation in anesthesia regards the education and training of anesthetic techniques for regional anesthesia, neuraxial blocks, and perioperative pain management.

The term extended reality (XR) refers to different modalities useful for immersive and interactive experiences. What unifies these methodologies is their fusion of the tangible realm (real-world) with digital components (digital world), ultimately resulting in a transformative alteration of reality. More properly, XR is an umbrella term that includes virtual reality (VR), augmented reality (AR), and mixed reality, as well as any technology that implements computer-generated realities. Briefly, VR envelops users within entirely virtual environments, effectively disconnecting them from the physical world. Utilizing specialized devices such as headsets and, at times, hand controllers, users can navigate and engage with these digital realms, frequently encountering a profound sensation of being present within the virtual setting. On the other hand, in AR, digital elements are overlaid in the real world, creating a blended environment where virtual objects coexist with the physical surroundings. Incorporating visualization technologies into AR expands the possibilities for creating engaging and interactive experiences that merge digital and physical elements seamlessly. MR combines elements of both VR and AR, allowing users to interact with virtual objects that appear to coexist with the physical world. A brief description of immersive XR technologies is presented in Table 4.

**Table 4:** Brief description of immersive extended reality (XR) technologies

**XR modality**	**Functioning**	**Features and properties**
Virtual reality (VR)	It immerses users within fully simulated environments, effectively disconnecting them from the physical world.	Through specialized devices (e.g., headsets and hand controllers) users gain the ability to explore and engage with these digital spaces, in a real-time interaction.
Augmented reality (AR)	It overlays digital content onto the tangible world via devices like smartphones, tablets, or AR glasses.	AR technology enables users to simultaneously perceive both their physical surroundings and the digital elements, resulting in interactive experiences that enhance real-world environments.
Mixed reality (MR)	It amalgamates the attributes of VR and AR, empowering users to interact with virtual objects that seemingly coexist with their physical environment.	MR fosters a heightened level of interaction and integration between digital and real-world components, reshaping the way people engage with their surroundings.

The foundational technology behind XR is undergoing rapid and continuous evolution. As computational power, hardware capabilities, and software sophistication progress, XR experiences are becoming more immersive, seamless, and captivating. In this complex scenario, high-quality 2D, and 3D approaches, and 360 media refer to advanced techniques used in creating visual content that offers enhanced realism, depth, and immersive experiences across different dimensions. For example, spherical virtual environments and 360-degree recordings represent an incredible technological improvement [39]. They create an all-encompassing visual experience where the users feel as if they are situated at the center of a sphere, surrounded by a complete view of their surroundings. These environments are typically developed using 360-degree cameras that capture real-world scenes from all directions simultaneously, allowing viewers to look in any direction during playback, or by stitching together multiple images or videos obtained from different angles. Users can explore these environments by using VR head-mounted displays (HMDs) such as virtual headsets, smart glasses, or other immersive devices. As they turn their head or move their body, the view adjusts accordingly, providing a sense of presence and immersion. Spherical virtual environments are extensively used in various applications, such as virtual tours of real-world locations, architectural visualization, training simulations, and entertainment experiences, allowing users to engage with content from every angle.

Due to technological advances in terms of miniaturization and improvement of processing capacity, immersive technologies, and computer-based simulations are being integrated into medical education, further enriching the training experience. The development process of immersive reality systems in the field of medicine is notably intricate and demands the synergistic collaboration of diverse professional skills and knowledge. This collaborative landscape includes medical experts, graphic designers, software developers, and user experience specialists, who work in tandem. Their collective efforts can harmonize the intricate technicalities with medical expertise. A project involving the development of AR or VR for ultrasound training necessitates a complex preliminary phase for the acquisition and definition of anatomical images. It requires the transformation of medical images into sophisticated 3D models. However, precision is mandatory to capture even the most intricate anatomical details, ensuring that the resulting models are not only accurate but also richly informative. Subsequently, these intricate 3D models undergo an intensive refinement process. Every aspect is scrutinized and adapted to guarantee an unparalleled level of accuracy and realism. Rigorous revisions are conducted, aligning with the highest industry standards to uphold the quality and authenticity of the final product.

**Fig. 2:**
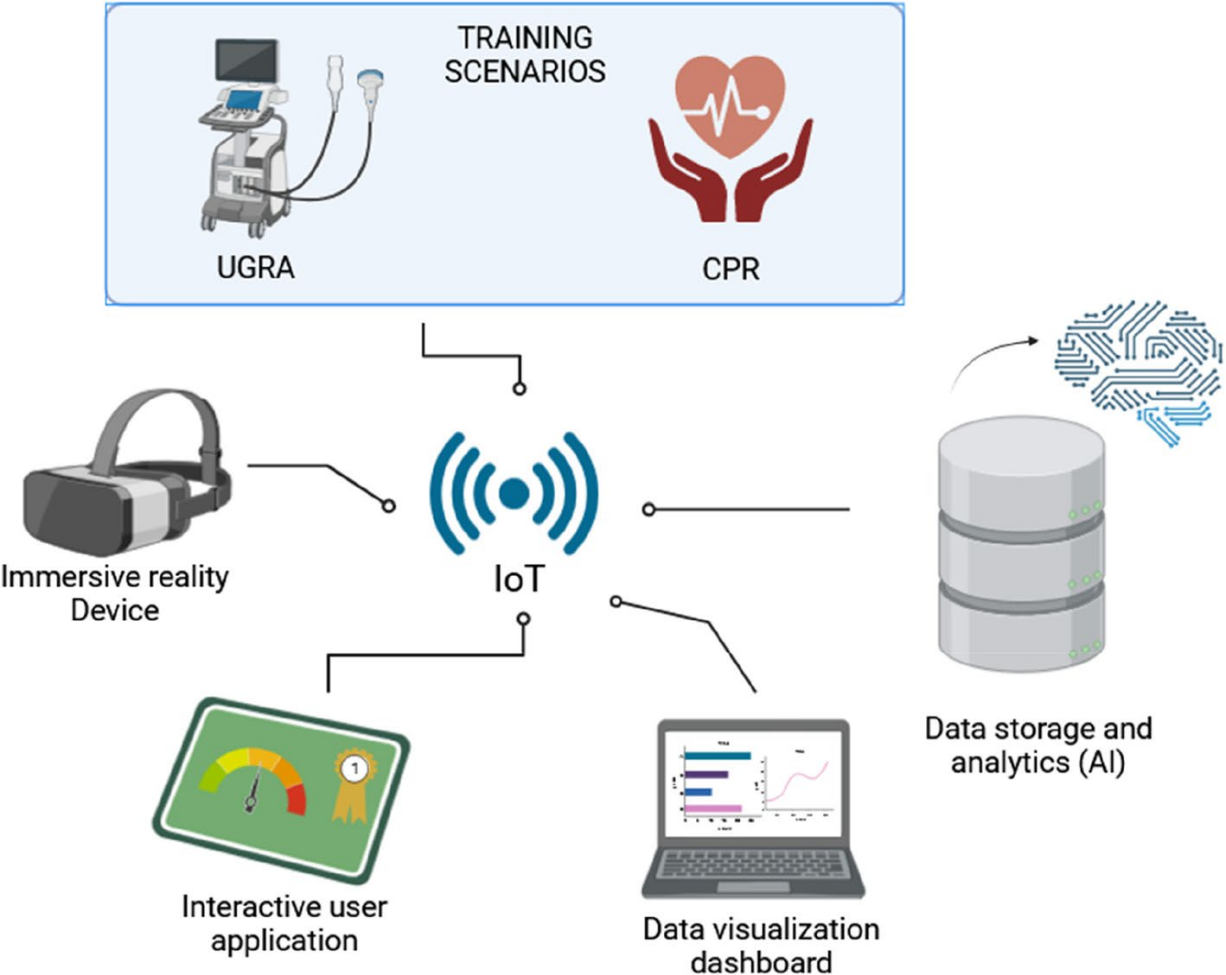
The convergence of immersive technologies, simulations, and visualization is catalyzing a revolutionary phase in education and training, including the field of anesthesia and critical care. By implementing Internet of Things (IoT) strategies, wearable components can be seamlessly integrated into gamified scenarios (two examples presented), yielding valuable data that can be stored and leveraged for artificial intelligence analysis. Abbreviations: IOT, Internet of Things; UGRA, ultrasound-guided regional anesthesia; CPR, Cardiopulmonary Resuscitation

Therefore, the fusion of immersive technologies, simulation, and visualization is ushering in a transformative era in education and training, even in anesthesia. Significantly, it can involve the utilization of wearable devices and Internet of Things (IoT) strategies (Fig. 2). This amalgamation is not merely a confluence of technologies, but a powerful synergy that promises to revolutionize the way for learning and acquiring skills. Immersive technologies can create interactive scenarios that allow learners to engage with the content in ways that were previously unimaginable. For instance, medical students can practice intricate surgical procedures in a risk-free virtual environment, refining their techniques and decision-making skills. Simulation serves as the backbone of experiential learning, replicating real-world situations and challenges. Visualization, meanwhile, complements these technologies by transforming complex data and concepts into visual representations that are easy to comprehend. Through interactive visualizations, abstract theories come to life, making it easier for learners to grasp intricate details and relationships.

#### Gamification and simulation in ultrasound-guided regional anesthesia

An interesting area of simulation and gamification application involves acquiring proficiency in ultrasound-guided regional anesthesia (UGRA). Simulation-based pathways have proven to enhance skill mastery in UGRA. Simulation models have incorporated various methodologies, such as phantoms, cadavers, web-based online learning, and dedicated sessions held within simulation centers. Nevertheless, although in 2010, the American Society of Regional Anesthesia and Pain Medicine and the European Society of Regional Anaesthesia and Pain Therapy (ASRA-ESRA) recommended the inclusion of UGRA curriculum with simulation training in residency programs, there is currently no established standard for simulation-based training methods in this domain [40].

The possibilities for innovation are intricately linked to advancements in AI technologies, given the extensive potential that AI holds in regional anesthesia. They span from creating sophisticated clinical decision support tools to analyzing performance metrics during simulation training. Ultimately, AI could pave the way for the creation of robots capable of enhancing needle tip precision and optimizing the administration of local anesthetics. In addition, computer vision, a subset of AI, leverages convolutional neural networks to adequately capture intricate spatial and temporal characteristics present in images (e.g., nerves and tissues) through the application of filtering and pooling methods [41].

Effectively conducting a UGRA procedure necessitates a combination of three interconnected proficiencies: capturing clear images of sonoanatomy, interpreting anatomical structures, and maintaining precise hand–eye coordination. AI-based immersive technologies can be adopted for all these aims. For instance, Chuan et al. [42] developed a custom VR training software utilizing advanced motion capture and ultrasound imagery at high resolution. Their software was designed to impart the cognitive-motor needling skills required for proficiently executing UGRA procedures. In this complex and fascinating scenario of simulation-driven training, educational tools like gamification can strongly promote competencies [43]. A review based on evidence demonstrated that simulation training led to significant enhancements in UGRA knowledge and skills, evaluated at levels 2 (knowledge and skills), 3 (application of learning to the workplace), or 4 (patient outcomes) of the Kirkpatrick scale [44].

#### Obstetric anesthesia training

Obstetric anesthesia training equips medical professionals with the specialized skills and knowledge required to ensure safe and effective anesthesia management during childbirth, addressing the unique challenges posed by labor and delivery scenarios. Within this domain, Gibiino et al. [13] undertook a study involving 282 anesthesia trainees from five Italian universities who were enrolled in the Online Obstetric Anesthesia Course (OOAC) and were provided with the FantaTraining ® app. These participants were divided into two groups at random: one enabled to engage in the league aspect of the app (study group), and the other not enabled (control group). All trainees underwent initial and concluding evaluations, each featuring 40 identical multiple-choice questions, administered before and after the completion of the OOAC course. The findings revealed no disparities in pre-course test scores between the groups. The mean score attained in the final test exceeded that of the initial test in both groups. However, the study group's final test scores significantly surpassed those of the control group (*p* < 0.001). More recently, Lee et al. [45] developed and tested the EmergenCSim™. It is a serious game incorporating an embedded assessment and a debriefing tool, aimed at instructing and evaluating the execution of general anesthesia for cesarean delivery in anesthesia trainees. Although the game-based approach did not lead to significant enhancements in the final quiz scores, its framework should be used to develop more articulated gamification pathways.

#### Game‐based distraction in adult and pediatric anesthesia

Preoperative anxiety is highly prevalent, affecting up to 80% of patients [46]. Common sources of this anxiety include concerns about the surgery itself, the anesthesia process, and potential complications (such as pain and nausea). Other contributing factors include past negative experiences with surgery or anesthesia, as well as individual predispositions. This issue also affects the pediatric population. The profound anxiety experienced by children during surgery can exert substantial and enduring effects on their physical and psychological health. It is noteworthy that nearly 20% of children might encounter stress-induced negative behavioral changes following surgery, resulting in impediments to timely recovery and challenges in self-care [47]. Furthermore, preoperative anxiety has been linked to heightened postoperative pain and escalated utilization of analgesics [48].

Game-based distraction techniques encompass a diverse range of methods. These include digital games, where electronic devices like tablets or smartphones are utilized; VR, immersing the child in a virtual world through headsets for interaction with digital environments during medical procedures; and interactive distraction, involving engaging the child with hands-on activities like toys and puzzles that demand focus and participation. Additionally, guided imagery employs storytelling or visualization to guide the child's imagination, creating a mental diversion from the medical procedure. Projected visuals utilize dynamic, colorful images or animations displayed on screens or walls within the medical environment to captivate the child's attention.

Among these methods, utilizing games-based and audio-visual interventions as distraction resources presents a novel approach that holds the potential to significantly alleviate perioperative pain and anxiety. For example, Dwairej et al. [49] showed that the integration of video game distraction alongside anesthesia mask exposure and shaping can offer a straightforward, safe, and time-efficient intervention to effectively mitigate children's anxiety and enhance their compliance during anesthesia induction. Additional research has explored the application of audio-visual distraction methods, utilizing elements like cartoons, video clips, interactive games, virtual reality, and humanoid robots, to alleviate distress among children undergoing painful procedures such as venous access, cancer therapy, or burn treatment [50, 51].

Interestingly, researchers have tested the synergistic power of gamification and VR in pediatric anesthesia. In a brief 5-min VR game developed by JSC GAMES (Seoul, Korea), pediatric patients were immersed in a comprehensive preoperative process and the induction of general anesthesia within a 360°, 3D virtual environment. This virtual experience incorporated gaming elements such as a virtual world, progression, exploration, challenges, and rewards. Within this virtual environment, players were virtually transported to the operating room, allowing them to interact with and explore the setting, including monitoring equipment. Upon selecting a device, players were presented with a detailed explanation of its function. Moreover, they were given the opportunity to learn the proper breathing technique using a facial oxygen mask. They could even choose their preferred fragrance for the mask. As players advanced through the game, they encountered challenges to conquer a “germ monster”. Each successful advancement earned them “health points” [14].

Results from evidence-based analyses addressing the topic of game-based approaches and anesthesia are very encouraging. In a recent systematic review and meta-analysis, the authors examined 26 studies involving a total study population of 2525 children. The review demonstrated that interventions based on gaming, whether through gamification or VR, effectively reduce preoperative anxiety in children [52].

#### Gamification for pain management

Gamification in the context of pain management is predominantly centered around app-based solutions. As Gonzales et al. [53] demonstrated, the incorporation of gamification has proven to be impactful in driving significant behavioral changes. Pieces of evidence demonstrated that telehealth-based approaches and mobile health (mHealth) strategies centered around pain management apps are reshaping the landscape of chronic pain management [54–56].

Given the heterogeneity of settings and the numerous issues revolving around the phenomenology of pain, the development of these methodologies is a complex task. To maximize their impact, chronic pain management apps should rest on evidence-based content, encompassing pain-related education, self-monitoring, goal setting, social support, and the instruction of self-care strategies, including stress management. Nevertheless, relying solely on evidence-based content has demonstrated limited ability to ensure robust user engagement and motivation. As highlighted by Jamison et al. [57], there is a call for improvements to amplify the appeal and entertainment value of pain management apps. Employing gamification can be a valid method to enhance user engagement and motivation. However, the inclusion of game elements in nongame scenarios within health apps has been the subject of a comprehensive evaluation, as its effectiveness seems to be closely tied to the distinct context and objectives of each app [7].

#### Other applications: perioperative training, avatars, and research

Gamification also holds the potential to motivate patients to calibrate pre-operative training programs. A variety of gamified health apps and wearable technologies have been released to effectively address specific conditions [58]. These methods have been also adopted to boost patient’s compliance with post-operative care guidelines, encompassing tasks such as adhering to medication timetables, engaging in prescribed exercises, and attending follow-up appointments [59]. Patients and physicians can collaboratively establish objectives aimed at enhancing physical, mental, social, and functional well-being. This approach can foster a heightened awareness of responsibility and commitment among patients, thereby increasing their propensity to engage proactively in the journey of recuperation. This aspect is closely intertwined with patient monitoring, involving applications designed to monitor users’ health status and track improvements or deteriorations in their lifestyles [60]. These games, such as the PERGAMON Platform for diabetes care [61], often include features such as competing with other users based on key performance indicators and sharing achievements between patients, peers, and their caregivers, in a secure way.

Interestingly, the combination of avatars and gamification can be seen in applications where patients interact with virtual avatars for perioperative training. For instance, virtual health coaches (avatars) can guide patients through exercises, dietary plans, and medication schedules using gamified approaches. These avatars can also provide real-time feedback and motivation to users, enhancing their overall health management experience [58].

Finally, gamification offers promising avenues in anesthesia research. By integrating simulation-based techniques with gamification, researchers can delve into a spectrum of areas, including the analysis of training efficacy and skill evaluation, enhancement of patient safety and overall quality of care, as well as the optimization of patient experiences and educational initiatives.

### Gamification design and cost challenges

Structuring a gamification process encompasses a diverse array of skills and requires careful investments to offset associated expenditures. These costs and resource allotments necessitate meticulous evaluation, considering their equilibrium against the projected advantages. In 2021, a market analysis found that estimated baseline costs may range between $45,000 and $60,000, excluding expenses for research, wireframing, storyboarding, and additional components such as HMDs for XR [62].

**Fig. 3:**
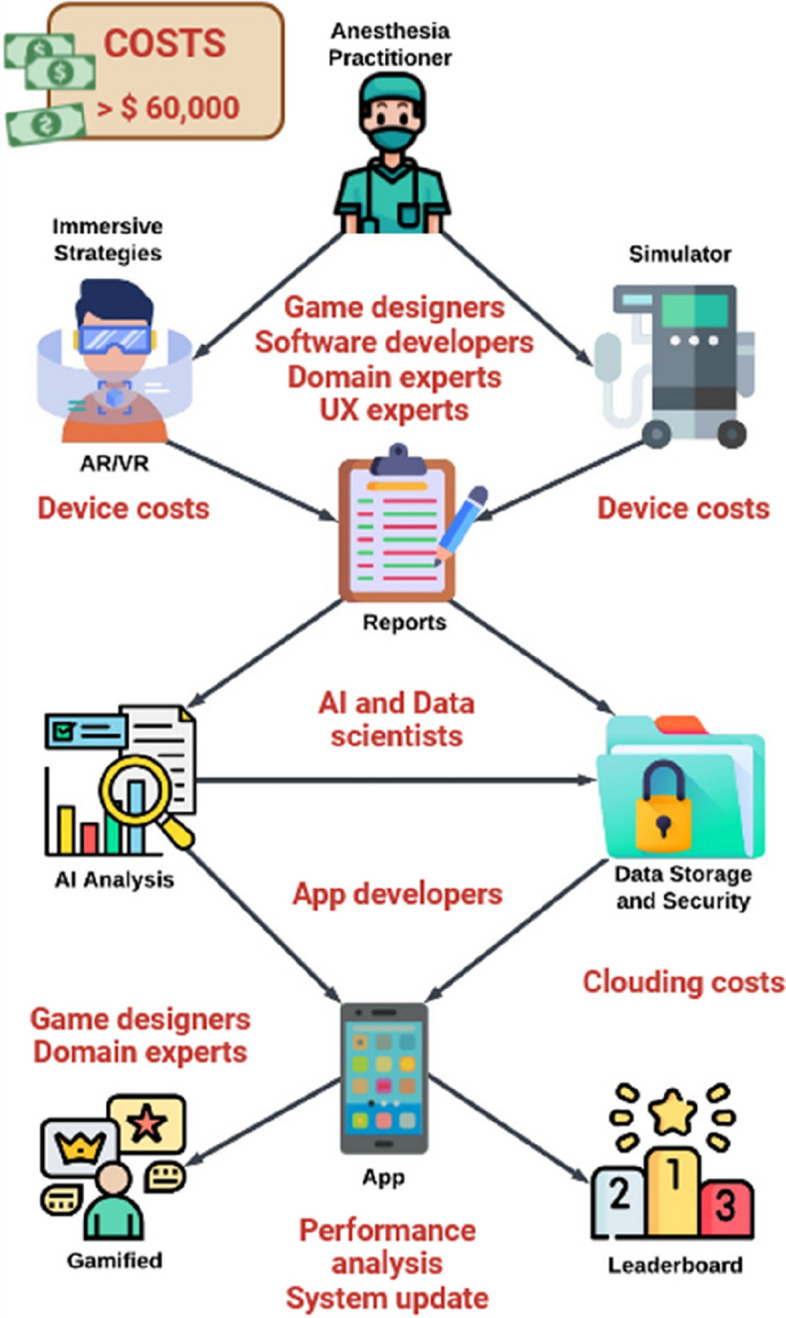
Development of a gamification process for anesthesia trainees. The estimated costs exceed $40,000 to $60,000. These costs increase with the acquisition of immersive reality technology. Several professional roles are involved. The list is not exhaustive. Abbreviations: AI, Artificial Intelligence; AR, augmented reality; VR, virtual reality; UX, user experience

The process of developing a gamification system for anesthesia trainees is highly complex. It mandates the proficiency of game designers, software and IT developers, user experience (UX) designers, anesthesia educators, specialized domain experts, and other professionals (Fig. 3).

The key step of the gamification design is the creation of the training platform and application. Another pivotal phase is the make-up of a user-friendly interface, inclusive of interactive modules and comprehensive progress tracking. Designing UX for XR and gamification is an intricate task demanding a range of skill sets. Seamless integration with existing simulation systems, along with a robust data repository, is also imperative. Equally significant is the design of game mechanics that harmonize with anesthesia training objectives. It must provide the collaborative efforts of game designers and content creators who formulate anesthesia-centric scenarios replete with challenges, rewards, and feedback mechanisms. The aim is to sustain user engagement while concurrently nurturing skill enhancement. Moreover, depending on the complexity of the gamification system, the requirement of dedicated hardware and devices such as XR tools must be considered. These tools can markedly augment the costs of the whole gamification process. The involvement of IoT experts is also needed to structure the network of physical objects or “things” that are embedded with sensors, software, and other technologies, enabling the collection and exchange of data over the Internet. Furthermore, the ongoing maintenance and updates of the system constitute another substantial investment. Finally, since gamification systems that handle patient data or medical records must adhere rigorously to stringent data security and privacy regulations, it becomes imperative to acknowledge and address potential expenses for encryption and secure data storage [63].

## Conclusion

In anesthesia, pain medicine, and critical care, there are numerous potential applications of gamification strategies for educational and training purposes, particularly when leveraging the augmented synergy between game-based methods and AI. The fusion of immersive technologies, simulation, and visualization is shaping a future where education and training attain unparalleled levels of immersion, engagement, and efficacy. In these complex clinical fields, the adoption of gamification strategies holds the promise of significant developments, encompassing both simulation systems and non-simulated scenarios. This dynamic approach has the capacity to yield noteworthy enhancements in knowledge retention, skill acquisition, and overall performance, improving both technical and non-technical skills. Interesting perspectives regard the use of gamification for optimizing patient comfort and compliance in the perioperative period and in different pain medicine settings. Finally, these strategies represent an incredible and underestimated resource for research applications.

## Data Availability

The datasets generated during the current narrative review are available from the corresponding author upon reasonable request.

## Abbreviations

AI: Artificial intelligence
IT: Information technology
VR: Virtual reality
AR: Augmented reality
XR: Extended reality
HMD: Head-mounted display
CPR: Cardiopulmonary resuscitation
CMR: Crisis resource management
POCUS: Point-of-care ultrasound
IoT: Internet of Things
UGRA: Ultrasound-guided regional anesthesia
UX: User experience
